# Video Consultation Use by Australian General Practitioners: Video Vignette Study

**DOI:** 10.2196/jmir.2638

**Published:** 2013-06-19

**Authors:** Moyez Jiwa, Xingqiong Meng

**Affiliations:** ^1^Curtin UniversityCurtin Health Innovation Research InstituteCurtin UniversityPerthAustralia; ^2^School of Public HealthCurtin UniversityPerthAustralia

**Keywords:** videoconferencing, general practice, patient appointments, health care

## Abstract

**Background:**

There is unequal access to health care in Australia, particularly for the one-third of the population living in remote and rural areas. Video consultations delivered via the Internet present an opportunity to provide medical services to those who are underserviced, but this is not currently routine practice in Australia. There are advantages and shortcomings to using video consultations for diagnosis, and general practitioners (GPs) have varying opinions regarding their efficacy.

**Objective:**

The aim of this Internet-based study was to explore the attitudes of Australian GPs toward video consultation by using a range of patient scenarios presenting different clinical problems.

**Methods:**

Overall, 102 GPs were invited to view 6 video vignettes featuring patients presenting with acute and chronic illnesses. For each vignette, they were asked to offer a differential diagnosis and to complete a survey based on the theory of planned behavior documenting their views on the value of a video consultation.

**Results:**

A total of 47 GPs participated in the study. The participants were younger than Australian GPs based on national data, and more likely to be working in a larger practice. Most participants (72%-100%) agreed on the differential diagnosis in all video scenarios. Approximately one-third of the study participants were positive about video consultations, one-third were ambivalent, and one-third were against them. In all, 91% opposed conducting a video consultation for the patient with symptoms of an acute myocardial infarction. Inability to examine the patient was most frequently cited as the reason for not conducting a video consultation. Australian GPs who were favorably inclined toward video consultations were more likely to work in larger practices, and were more established GPs, especially in rural areas. The survey results also suggest that the deployment of video technology will need to focus on follow-up consultations.

**Conclusions:**

Patients with minor self-limiting illnesses and those with medical emergencies are unlikely to be offered access to a GP by video. The process of establishing video consultations as routine practice will need to be endorsed by senior members of the profession and funding organizations. Video consultation techniques will also need to be taught in medical schools.

## Introduction

Australia is a geographically dispersed country in which one-third of the population lives in rural and remote locations. Inequity in health care is thought to be linked to the cost of medical appointments and to the shortage of medical manpower in rural and remote areas [1,2]. In other sectors, access to services has been facilitated by information technology. For example, there is growing evidence for the role of information technology to improve the customer experience in the retail and finance industries [3,4].

In theory, access to doctors can also be efficiently facilitated by online video technology [5]. However, video technology requires both practitioners and patients to be willing to consult via the Internet. To date, the deployment of online video technology in Australian primary care is not routine practice. The practice is limited to government-subsidized consultations involving specialist practitioners or to small numbers of privately funded schemes [6,7].

People who consult doctors in general practice are heterogeneous [8]. The reasons for seeking medical advice range from self-limiting conditions of recent onset to chronic and life-limiting problems [8]. The symptoms or problems presented may warrant information, education, reassurance, explanation, examination, prescription, referral, and/or investigations. The consultation provides an opportunity to address the patient’s current problems, and also to consider and potentially prevent future problems [9].

In a face-to-face consultation, the doctor can use all 5 senses; however, in an Internet-based video consultation access to sensory information is limited, and the information that is available may be hampered by download speeds and/or the performance of computer hardware. Furthermore, there is no scope to intervene in person if the patient requires immediate resuscitation. Many of these limitations also apply to telephone consultations; yet, in parts of the world telephone consultations in primary care are considered routine and time saving [10,11].

This Internet-based pilot study aimed to explore Australian general practitioners’ attitudes to video consultation with a range of patients who may not be known to them previously. The survey tool used in this study was based on the theory of planned behavior (TPB) [12].

## Methods

This study was approved by the Curtin Human Research Ethics Committee (No: RD-61-12).

Participants were recruited from members of the Curtin Health Innovation Research Network (CHIReN), a virtual network of general practitioners (GPs) across Australia who have already consented to be invited to participate in studies with standardized patients. The study took place over approximately 12 weeks. Each participant was remunerated AUS $50 as recompense for his/her time to participate in the study.

**Figure 1:**
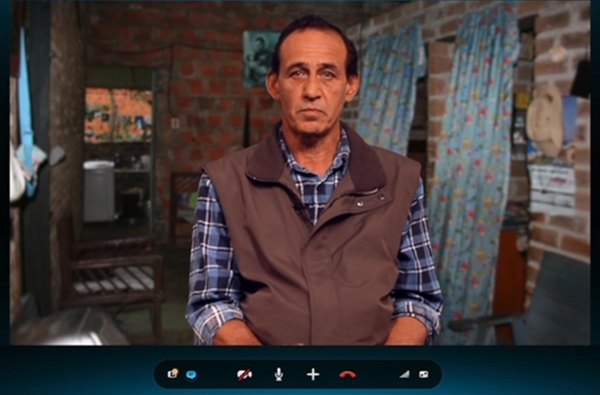
Video consultation vignette.

Participants answered questions after viewing video-recorded monologs by actor-patients. Video scenarios were produced and validated by a team of 6 GPs. Six videos were produced, each featuring an actor-patient presenting a range of clinical problems. Information on medical history, family history, and drug history were offered at the outset of each video. The range of scenarios is consistent with those reported in the GP activity reports [8,13]. Scenarios are described in Table 1. A screenshot from 1 of the videos is shown in Figure 1. The vignettes ranged from a self-limiting minor illness to a life-threatening medical emergency. Participating GPs provided their demographic details and answered questions about their impressions (see Table 2).

**Table 1:** Scenarios presented to participants in videos.

Video vignette	Patient information	Description of condition
1	Patient: Fay Connolly. Occupation: dental nurse. Age: 49. Nonsmoker. Alcohol: 2 units per week at most	History: Anxiety and depression for 3 months. Has refused antidepressants in the past, now struggling to cope. Having recurrent panic attacks, can’t sleep at night. No energy, loss of libido, can’t concentrate for any length of time. Tearful. Wants help. Not sure can keep going to work anymore. Last consultation June 2011: Bit anxious and depressed, referred to counselor-did not attend appointments. Husband is very worried
2	Patient: Lucy Jones. Occupation: unemployed. Age: 51. Smoker: 30/day. Alcohol: 6 units per week (as recorded in 2008)	History: Abnormal liver function tests-drinking half bottle of vodka every day. Started drinking after divorce 4 years ago, a few glasses of wine 3-4 times a week, now drinking steadily every day. Was stopped by police 1 week ago and facing court appearance for driving while intoxicated. Son is at university and now refusing to visit his mum. Was found sleeping in her own vomit in the bathroom 1 week ago. Spending most of her money on alcohol, rent hasn’t been paid for 2 months. Feels depressed most of the time. Not eating well, tired all the time. Last consultation June 2010: upper respiratory tract infection
3	Patient: Adrian Marshall. Occupation: store room supervisor. Age: 49	History: Recurrent bouts of crushing chest pain. Started at 3 am this morning and now has been present for several hours. Nothing seems to help. Has vomited several times; feeling a bit breathless. Pain is worse when moving about, but now severe even when sitting quietly. Feeling a bit dizzy. Been sweating a bit. Feeling very worried. Past history: hypertension on Ace inhibitor since 2006. Last blood pressure recorded 6 months ago: 155/96 mmHg. Smoker 20/day. Wife is very worried. Seen on video with arm around patient
4	Patient: Nellie O’Reilly. Occupation: receptionist. Age: 59. Nonsmoker. Nondrinker	History: Recurrent bouts of central abdominal pain for 2 months, has had 6 attacks so far. No specific pattern to the symptoms, can occur at any time, pain is mainly on the right-hand side and can last 2 hours. Occasionally vomits during an attack. Left with dull ache after each episode. Sometimes feels like she is wearing a tight band around her upper abdomen and some aching of right shoulder. Last attack was 2 days ago in the middle of the night. Son offered to drive her to hospital but by the time he arrived at her house the pain was gone. Worried it might be something serious. Last consultation September 2011: upper respiratory tract infection. Advised over the counter analgesia and fluids
5	Patient: Richard Cunningham. Occupation: Truck driver. Age: 48. Smoker: 20/day	History: Diabetes mellitus on biguanide for 6 months. Blood pressure: 156/80 mmHg (3 weeks ago), previous readings: 150/85 mmHg (3 months ago). Diet: poor. Body mass index: 30 Cholesterol: 6.7 mmol/L, LDL: 2.5 mmol/L. HbA1C: 7.5 mmol/L. Needs repeat medication: Biguanide and calcium channel blocker. Last consultation November 2011: attended for driving license medical
6	Patient: Mary Smith. Occupation: librarian. Age: 60. Nonsmoker	History: Cough and sore throat for 3 days. Doesn’t feel unwell otherwise, but can’t manage at work because she needs to use her voice. Cough keeping her awake at night. Current medication: Ace inhibitor for hypertension for the past 2 years. No allergies. Wants an antibiotic-always seems to help. Last consult August 2011: upper respiratory tract infection. Prescribed: antibiotic

**Table 2:** Questions for participants after each video.

Domain	Question	Question type	Options/range
	1. What is your differential diagnosis?	Free-text comment	
**Intention**			
	2. Would you continue with this consultation online?	Choose 1 option	Yes/no/maybe
	3. How difficult was it for you to suggest a diagnosis for this scenario?	7-point scale	Not at all difficult to very difficult
**Attitude**			
	4. Managing a patient like this online is:	7-point scale	Harmful to beneficial
		7-point scale	Worthless to useful
		7-point scale	Not convenient (for me) to convenient (for me)
**Subjective norm**			
	5. Most patients/GPs/specialists think I ___ consult people like this online	7-point scale	Should to should not
	6. Medicare thinks I ___ consult people like this online	7-point scale	Should to should not
	7. It is expected of me that I should consult patients like this online	7-point scale	Strongly agree to strongly disagree
	8. I feel under social pressure to consult patients like this online	7-point scale	Strongly agree to strongly disagree
**Perceived behavior control: self-efficacy**			
	9. I am confident that I could consult patients like this online if I wanted to	7-point scale	Strongly agree to strongly disagree
	10. For me to consult patients like this online is:	7-point scale	Easy to difficult
**Perceived behavior control: controllability**			
	11. The decision to consult patients like this online is beyond my control	7-point scale	Strongly agree to strongly disagree
	12. Whether I consult online is entirely up to me	7-point scale	Strongly agree to strongly disagree

### Theory of Planned Behavior

This theory postulates that a person’s behavior is determined by his/her intention to perform the behavior. This intention is determined by their attitude toward the specific behavior, their subjective norms, and their perceived behavioral control. These 3 domains were explored in this study with reference to scenarios depicting clinical challenges regularly presented to GPs in Australia [13]. The items of the TPB, as measured in this study, were as follows, and each is taken from a guide to the development of such questionnaires [14].

#### Intention

The respondents were presented with a video scenario and asked whether they would continue with the video consultation. They were also asked to suggest the level of difficulty of making a diagnosis on a scale from 1 to 7, with 1 being not at all difficult and 7 being extremely difficult. The higher the number, the stronger the intention to perform the behavior. For diagnosis difficulty, we calculated the mean of responses for each participant (which may modify the relationship between intention and actual behavior) or the mean for all participants across each scenario (which may reflect differences between scenarios).

#### Attitude

Direct measurement of attitude involved the use of bipolar adjectives (ie, pairs of opposites), which are evaluative (eg, good–bad). We calculated the mean of the item scores to give an overall attitude score. The attitude items were also scored for internal consistency (Cronbach alpha=.88).

#### Subjective Norms

Direct measurement involves the use of questions referring to the opinions of important people in general. The subjective norm items were scored for internal consistency (Cronbach alpha=.87). We calculated the mean of the item scores to give an overall subjective norm score.

#### Perceived Behavior Control

This was achieved by assessing the respondent’s self-efficacy and their beliefs about the controllability of the behavior. Self-efficacy was assessed by asking people to report how (1) difficult it was to perform the behavior, and (2) confident they were that they could do it. Controllability was assessed by asking people to report whether (1) performing the behavior was up to them, or (2) factors beyond their control determined their behavior.

### Scoring

We checked that the subjective norm items had high internal consistency (self-efficacy: Cronbach alpha=.84; controllability: Cronbach alpha=.72). We calculated the mean of the item scores of the 2 questions related to self-efficacy (questions 9 and 10 in Table 2), and the mean of the item scores of the controllability-related questions (questions 11 and 12 in Table 2) to give mean scores for self-efficacy and controllability. The mean scores of the 2 self-efficacy questions and the 2 controllability questions were also calculated to give an overall perceived behavioral control score (Cronbach alpha=.58). Scores for questions 9, 10, and 12 were reversed before calculation, so that high scores consistently reflected a greater level of control over the target behavior.

### Analysis

We confirmed that all internal consistency coefficients were acceptable (> 0.7); therefore, it was appropriate to include all the items in the composite variables. Using a multiple regression procedure, we entered intention as the dependent variable, and the direct measures of attitude, subjective norm, and perceived behavioral control (self-efficacy and controllability) as the predictor variables.

### Sample Size

A sample of 47 to 62 GPs would give us 80% power to reject the null hypothesis; that is, 50% of GPs would proceed with the consultation in most cases if the true proportion of GPs who choose to proceed was 70%. Such proportions are consistent with previous research [15].

### Data Collection and Analysis

Multivariable logistic regression was used to determine if any identifiable subgroups of GPs, according to demographic criteria, showed significant differences in their scores. *P* values less than .05 were considered statistically significant. Stata version 12.1 (StataCorp LP, College Station, TX, USA) was used to perform the analysis. Multiple regression models were adjusted for the lack of independence between individual participants by estimating the clustered standard errors to account for intragroup correlation (vce option in Stata).

## Results

Forty-seven GPs were recruited from the 102 that CHIReN has on file, which is a response rate of 47%. One general practitioner omitted all demographic questions and was excluded from the analysis, resulting in a total sample of 46. The demography of the sample is reported in Table 3, which also demonstrates a few significant differences between the participants and the known average profile of GPs in Australia.

The GPs offered a differential diagnosis for each video and the level of difficulty in making a diagnosis for that scenario. Data in Table 4 demonstrate that scenario 4 (patient with gall bladder disease) was the most difficult to diagnose, closely followed by scenario 2 (patient with alcoholism) and scenario 6 (patient with an acute cough).

Table 5 summarizes the participant’s intention to continue with the consultation. Respondents were least likely to continue with the video consultation with the patient who appeared to be having chest pain, and most likely to continue with the patient seeking a repeat prescription for diabetes and hypertension.

Data relating to the TPB are presented in Table 6. A range of views were expressed with further comments presented subsequently. For the current study, the results show that GPs’ self-efficacy and controllability toward online consultations are not in the same direction. When calculating the overall Cronbach alpha for the behavior control score, the 2 controllability scores are in the opposite direction (negative direction) of the 2 self-efficacy scores (positive direction). Therefore, we report self-efficacy and controllability separately. Although the overall behavior score was calculated and reported, it was not used in the regression model as a predictor because of the internal consistency of 4 items.

The relative risk (RR) ratio of difficulty of diagnosis and TPB scores associated with the GPs’ intention to continue the consultation within each scenario is presented in Table 7. The ambivalent views and negative views are compared to positive views in multinomial logistic regression. Table 8 presents the results of regression analysis with intention to continue to consult as the dependent variable and scenarios and GP demographic factors as predictive variables. Results in the table are relative risks ratios for the groups who said that “maybe” or they “will not” continue the consultation compared with those who answered yes (RR=1). Results are derived from 1 multinomial logistic regression. Only significant variables, those with *P*<.05, were retained in the final model and reported. Table 9 presents the comments to each scenario as recorded by the participants after each scenario.

**Table 3:** Demography of participating general practitioners compared to nationally reported group data (where available).

Participants’ details		Participant numbers n=46	National group
Age (years), mean (SD)		42 (11)	50.5^a^
Gender (male), n (%)		26 (57)	56%^b^
**Qualifications and experience**			
	Years since graduation, mean (SD)	18 (11)	No data
	Years working as GP, mean (SD)	13 (11)	No data
	Number of GPs in the clinic, mean (SD)	7 (4)	≥7 (29%)^b^
	GP sessions/week, mean (SD)	7 (3)	No data
	GP registrar/GP in training (yes), n (%)	8 (17)	1000 (3.8%)^c^
	FRACGP (yes), n (%)	29 (63)	54%^b^
	Accredited (yes), n (%)	45 (98)	91%^b^
**Position, n (%)**			
	Principal	9 (20)	No data
	Nonprincipal	30 (65)	No data
	Other	7 (15)	No data
**State, n (%)**			
	New South Wales	5 (11)	31.6%^d^
	Queensland	4 (9)	17.7%^d^
	Victoria	12 (26)	26.2%^d^
	South Australia	2 (4)	9.2%^d^
	Tasmania	1 (2)	2.4%^d^
	Western Australia	21 (46)	10%^d^
	Australian Capital Territory	1 (2)	1.8%^d^
**Region of the clinic, n (%)**			
	Capital	21 (46)	No data
	Other metropolitan	18 (39)	No data
	Large rural	2 (4)	No data
	Small rural	3 (7)	No data
	Remote center	2 (4)	No data
**Practice details, n (%)**			
	Major cities	32 (70)	71%
	Inner regional	6 (13)	No data
	Outer regional	3 (6)	No data
	Remote	5 (11)	No data
**Country where graduated university, n (%)**			
	Non-Australia	14 (30)	
	Australia	32 (70)	67%
**Patients seen/week, n (%)**			
	<100	20 (43)	No data
	100-149	17 (37)	No data
	150-199	9 (20)	No data
**Direct patient care hours/week (hours), n (%)**			
	<11	4 (9)	
	11-20	5 (11)	11%^b^
	21-40	30 (65)	56%^b^
	41-60	7 (15)	33%^b^
**Non-English consultations, n (%)**			
	No	35 (76)	
	Yes, less than 25%	10 (22)	24%^b^
	Yes, more than 50%	1 (2)	

^a^[16]

^b^[13]

^c^[17]

^d^[18]

**Table 4:** Diagnosis and rating for each video (N=46).

Video	Most common diagnosis		Number of differential diagnosis		Level of difficulty in making a diagnosis (scale 1 to 7)	
	Diagnosis	%	Mean (SD)	Median (IQR)	Mean (SD)	Median(IQR)
1	Anxiety and depression	71.7	2.5 (1.2)	2.0 (1)	2.2 (1.3)	2.0 (2)
2	Alcoholism	95.7	3.0 (1.4)	3.0 (2)	3.3 (1.9)	3.0 (3)
3	Myocardial infarction	82.6	2.6 (1.7)	2.0 (3)	1.6 (1.1)	1.0 (1)
4	Gall bladder disease	93.5	2.7 (2.0)	2.0 (2)	3.6 (2.0)	3.0 (4)
5	Diabetes mellitus plus hypertension	78.3	2.3 (1.1)	2.0 (1)	1.9 (1.1)	2.0 (1)
6	Upper respiratory tract infection	100	1.9 (1.1)	2.0 (1)	3.2 (1.8)	2 (3)

**Table 5:** Comments made by GPs regarding intention of continuing with each video consultation (N=46).

Video	Most common diagnosis	Intend to continue with consultation, %		
		Yes	Maybe	No
1	Anxiety and depression	31	41	28
2	Alcoholism	15	48	37
3	Myocardial infarction	2	7	91
4	Gall bladder disease	20	24	56
5	Diabetes, plus hypertension	41	35	24
6	Upper respiratory tract infection (URTI)	17	50	33

**Table 6:** Participants’ response as per the domains of TPB per scenario (N=46).

Scenario	TPB, mean (SD)				
	Attitudes	Subjective norms	PBC control efficacy	PBC control controllability	Behavioral score^a^
Anxiety and depression	4.3 (1.1)	2.9 (1.0)	3.8 (1.7)	5.3 (1.8)	4.6 (1.1)
Alcoholism	3.8 (1.3)	2.7 (1.1)	3.3 (1.6)	5.5 (1.7)	4.4 (1.1)
Myocardial infarction	2.3 (1.6)	1.9 (1.3)	2.3 (1.7)	5.5 (1.6)	3.9 (1.1)
Gall bladder disease	3.4 (1.6)	2.7 (1.1)	3.3 (1.7)	5.4 (1.7)	4.4 (1.2)
Diabetes plus hypertension	4.5 (1.6)	3.6 (1.3)	4.6 (1.6)	5.5 (1.7)	5.0 (1.2)
URTI	3.9 (1.6)	3.6 (1.3)	4.0 (1.6)	5.5 (1.7)	4.8 (1.1)

^a^Mean score of the 2 PBC subcategories.

**Table 7:** The relative risk (RR) ratio of difficulty of diagnosis and TPB scores associated with GPs’ intention to continue the consultation within each scenario (N=46).

Risk and TPB		Scenario, RR (95% CI)^a^				
		Anxiety	Alcoholism	Gall bladder disease	Diabetes	URTI
**Of saying "maybe" vs "yes"**						
	Difficulty	0.4 (0.2-1.0)	0.4 (0.2-1.0)	0.9 (0.3-2.8)	0.7 (0.3-1.8)	25 (0-100)
	Attitudes	0.2 (0.1-0.7)^b^	0.3 (0.1-0.9)^b^	0.3 (0.1-0.9)^b^	0.4 (0.2-0.97)^b^	0.1 (0-0.7)^b^
	Subjective norms	5.3 (1.1-25.7)^b^	1.8 (0.6-5.2)	1.1 (0.4-3.0)	0.6 (0.2-1.9)	0.3 (0-2.6)
	Self-efficacy	0.8 (0.4-1.8)	0.9 (0.5-1.7)	2.3 (0.8-6.8)	0.9 (0.4-1.9)	1.3 (0.5-3.5)
	Controllability	0.9 (0.6-1.4)	1.2 (0.7-2.1)	1.1 (0.5-2.4)	0.9 (0.5-1.4)	0 (0-100)
**Of saying "no" vs "yes"**						
	Difficulty	0.4 (0.2-1.0)	0.4 (0.2-0.9)^b^	1.1 (0.4-3.4)	0.7 (0.2-2.0)	100 (0-100)
	Attitudes	0.2 (0- 1.1)	0.3 (0.1- 0.8)^b^	0.1 (0-0.4)^d^	0.3 (0.1-0.9)^b^	0.1 (0-0.6)^b^
	Subjective norms	6.0 (1.1-31.9)^b^	1.0 (0.4-3.0)	2.5 (0.7-8.8)	0.3 (0.1-1.3)	0.1 (0-1.3)
	Self-efficacy	0.2 (0.1-0.6)^c^	0.5 (0.3-1.1)	1.0 (0.3-2.8)	0.8 (0.3-2.0)	1.0 (0.3-3.4)
	Controllability	0.8 (0.4-1.6)	1.5(0.8-2.5)	1.0 (0.4-2.5)	1.3 (0.7-2.6)	0 (0-17.8)

^a^For the groups who said that they “maybe” or “will not” continue the consultation compared with those who answered yes (RR=1). Results are derived from 6 multinomial logistic regressions according to the scenario; result values greater than 100 are truncated to 100. Myocardial infarction is not reported because only one 1 participant said yes and 3 participants said maybe. Due to such small numbers in some categories, it was not possible to include that scenario in the model.

^b^ *P*<.05

^c^ *P*<.01

^d^ *P*<.001

**Table 8:** Sociodemographic and scenarios as indicators associated with the intention to continue the consultation online.

Factors modeled		RR (95% CI)
**Saying "maybe" compared to "yes"**		
	Scenario (anxiety, RR=1)	
	Alcoholism	2.9 (1.1-7.7)^a^
	Myocardial infarction	2.6 (0.3-26.0)
	Gall bladder disease	0.9 (0.3-3.1)
	Diabetes	0.5 (0.2-1.4)
	URTI	2.6 (0.8-8.8)
	Country of university (non-Australian, RR=1)	6.8 (1.8-25.2)^b^
	Years after graduation	1.4 (1.2-1.7)^c^
	Years as a GP	0.8 (0.7-0.9)^c^
	GP registrar (“no,” RR=1)	0.8 (0.2-3.1)
	Clinic remoteness	0.6 (0.4-1.1)
	Number of GPs	0.9 (0.7-1)a
	Nonprincipal, principal/others (RR=1)	6.5 (1.8-22.8)^b^
	Hours practiced/week	4.5 (2.0-9.9)^c^
**Saying "no" compared to "yes"**		
	Scenario (anxiety, RR=1)	
	Alcoholism	3.2 (1.2-8.5)^a^
	Myocardial infarction	74.2 (7.9-695.7)^c^
	Gall bladder disease	3.9 (1.0-13.0)^a^
	Diabetes	0.5 (0.2-1.5)
	URTI	2.4 (0.7-7.8)
	Country of university (non-Australian, RR=1)	11.0 (3.1-39.8)^c^
	Years after graduation	1.2 (1.0-1.5)^a^
	Years as a GP	0.8 (0.7-0.98)^a^
	GP registrar (no, RR=1)	0.3 (0.1-0.96)^a^
	Clinic remoteness	0.5 (0.3-0.9)^b^
	Number of GPs	0.9 (0.8-0.98)^a^
	Nonprincipal, principal/others (RR=1)	1.9 (0.6-5.5)
	Hours practiced/week	2.6 (1.3-5.2)^b^

^a^ *P*<.05

^b^ *P*<.01

^c^ *P*<.001

**Table 9:** Free-text comments per scenario.

Video (most common diagnosis)	Continue with video consultation?		
	Yes (n)^a^	No (n)^a^	Maybe (n)^a^
Anxiety and depression	“A lot could be sorted out for her online”(7)	“An online consultation would not be ideal as it may be harder to establish rapport” (3)	“Needs to be examined”(3)
Alcoholism	“This is another case where an initial treatment plan could be made online.” (3)	“...the use of an online consultation in this case inhibits developing rapport particularly with a patient whom I have only seen occasionally” (2); “Needs physical examination and probably blood tests”(4)	“Needs physical examination” (11)
Myocardial infarction	“Depending on how far away he is from me, I would either go to him now after calling an ambulance to him, or if he is too far way, I keep talking to him after calling an ambulance to take him to hospital” (1)	“Needs to call an ambulance urgently” (32)	“Cardiac chest pain must come to surgery or to ED” (1)
Gall bladder disease	“Would like to see her for follow-up consultation for examination”(6)	“Needs examination” (5)	“Requires examination, cannot be achieved online” (23)
Diabetes plus hypertension	“Most of the issues in this consult could be managed online quite effectively” (10)	“Could be a convenient way of discussing results, however this would be variable upon the results. Still has issue of being unable to examine” (12)	“Would like to see him in person to reinforce the importance of the control of diabetes, quitting smoking, cholesterol and blood pressure” (3)
URTI	“I would not necessarily prescribe antibiotics. There are no symptoms that make me concerned about a chest problem” (2)	“It is very hard to manage this case online without physical exam” (10)	“You would have a lot of difficulty justifying to the patient why you have not prescribed antibiotics, when you have not examined the patient” (4)

^a^Number in brackets represents number of participants making similar comments.

## Discussion

General practitioner participants in this study might conduct a video consultation with patients other than those presenting with what could be an acute life-threatening emergency. Participants formed 3 approximately equal groups: those who would continue with the video consultation, those who were ambivalent, and those who would not. The scenario involving the person with anxiety evoked this typical response. GPs who had qualified from an Australian university were more likely to be equivocal about video consultations. Similar opinions were expressed by those medical practitioners who had been qualified for longer. However, those who had been in general practice for longer and those who worked in group practices were more likely to favor video consultations.

Compared with the case of the patient presenting with anxiety and depression, GPs were more likely to reject continuing with a video consultation with the patient with alcohol dependence, myocardial infarction, or gall bladder disease. Their objections focused primarily around the inability to physically interact with the patient. Those who had been qualified as a medical practitioner for longer and those who worked longer hours were more likely to express negative views. On the other hand, participants who had been practicing as GPs for longer, GP registrars, those who worked in remote practice, and those from larger group practices were less likely to be negative about video consultations.

Practitioners who were ambivalent about continuing with video consultations expressed the view that it was difficult to diagnose the patient presenting with symptoms of a cough, albeit an upper respiratory tract infection. By contrast, they had positive views about managing patients online for all but the chest pain scenario. They were also concerned about the attitude of significant others, such as patients, colleagues, and funders, about conducting video consultations particularly in the case of the patient with anxiety and depression. With regard to the low consistency between self-efficacy and controllability, we postulated that GPs did not feel they were able to conduct video consultations even if they wanted to because such consultations are not subsidized by government funding. However, they felt confident about their ability to conduct video consultations.

GPs are unlikely to offer video consultations for patients with a minor self-limiting illness of recent onset. The reticence could be ascribed to the perceived need for a physical examination. The importance of clinical examination to establish a diagnosis for acute cough has been emphasized in previous literature [19]. However, a recent review suggests that physical signs, if present, have poor predictive value to detect infections that may benefit from antibiotics [20]. It is also highly unlikely that practitioners would consider video consultations for patients who have a life-threatening medical emergency. This may stem from the perception of an increased risk of failing to make an appropriate diagnosis in this context and the need for immediate resuscitation of a patient with cardiac chest pain [21,22]. Consultations for patients with chronic mental health issues may also be hampered unless there are clear indications that such consultations are approved by colleagues and funding agencies.

### Limitations

The practitioners who participated in this study were generally younger than most Australian GPs. They were also more likely to be registrars and those working in a larger practice than average. Although the geographical distribution included a representative sample of practitioners from rural and remote areas, there were fewer from some states in Australia. We also acknowledge that the doctors had no opportunity to ask questions or seek clarification from the actor-patients. This was a significant issue, although it would have been difficult within the limitations of technology and the resources available to allow for such interactions in circumstances in which practitioners across Australia, living in different time zones, wished to participate in their own time and actors were only funded to perform a single vignette. Experience from previous studies with live consultations between actors and practitioner were that more limited numbers of practitioners were able and willing to participate. This introduces bias and less generalizable results [23]. Secondly, we did not interview the practitioners to explore their perspective on the consultations. Our comments are, therefore, limited to their responses to a questionnaire. In these circumstances, we can only draw limited conclusions that domains of the theory of learned behavior offer a recognized theoretical grounding to frame the conclusions. The free-text comments also provide further information on the impressions of the practitioners.

### Strengths

The greatest strength of this study was the use of Internet-based video vignettes to gauge GP opinion. Video vignettes have significant advantages over other data collection methods, in particular, the advantage of realism, or something closely approximating it [23-25]. Video vignettes can present patient information to clinicians in a way that closely resembles their usual consultations. More understanding is generated in this way than by surveys on usual practice. In the context of this study, video consultations are not yet routine practice; therefore, it was important to present the practitioners with examples of scenarios. Video scenarios can simulate clinicians’ usual working environments and generate a range of typical responses from them in a way that questions asked in the absence of an example scenario or in relation to a text-based scenario cannot. Data collected are likely to be valid. Such simulations could also be used to introduce video consultations to students at medical schools.

### Conclusions

Australian GPs may adopt video consultations in their practice, but this is likely to be in larger practices with more established GPs, especially in rural areas. It is also likely that access to video consultations will need to focus on follow-up consultations, where the purpose of the consultation is not primarily to establish a diagnosis. Patients with minor self-limiting illnesses and those with medical emergencies are unlikely to be offered access to a GP by video. Medical practitioners appear confident about their ability to conduct video consultations; however, the process of establishing video consultations as routine practice will need to be endorsed by patients, members of the profession, and funding organizations. Video consultation techniques will also need to be taught in medical schools. Future research on this topic could follow a similar outline with vignettes, but include more interactive video consultations between practitioners and patients.

## Multimedia Appendix 1

Video vignette alcohol dependence.

## Multimedia Appendix 2

Video vignette sore throat and cough.

## Multimedia Appendix 3

Video vignette diabetes.

## Multimedia Appendix 4

Video vignette chest pain.

## Multimedia Appendix 5

Video vignette anxiety.

## Multimedia Appendix 6

Video vignette gall bladder symptoms.

## Abbreviations

CHIReN: Curtin Health Innovation Research Network
GP: general practitioner
RR: relative risk
TPB: theory of planned behavior
